# Aortic valve and vascular calcium score in pre-TAVI CT: correlation with early post-procedural complications

**DOI:** 10.1007/s11547-023-01603-y

**Published:** 2023-02-14

**Authors:** Sabrina Maria Milo, Patrizia Toia, Federico Midiri, Luigi D’Alessandro, Giulia Sollami, Aldo Panci, Vincenzo Pernice, Francesco Violante, Khalil Fattouch, Giuseppe Cutaia, Giuseppe Salvaggio, Giuseppe Lo Re, Emanuele Grassedonio, Massimo Galia, Ludovico La Grutta

**Affiliations:** 1 Department of Biomedicine, Neurosciences and Advanced Diagnostics-BIND, University of Palermo, Via del Vespro 127, 90100 Palermo, Italy; 2 GVM Care and Research, Department of Diagnostic Imaging, Maria Eleonora Hospital, Palermo, Italy; 3 GVM Care and Research, Department of Hemodynamics and Interventional Cardiac and Vascular, Maria Eleonora Hospital, Palermo, Italy; 4 GVM Care and Research, Department of Cardiovascular Surgery, Maria Eleonora Hospital, Palermo, Italy; 5 Department of Health Promotion, Mother and Child Care, Internal Medicine and Medical Specialties - ProMISE, University of Palermo, Via Del Vespro 12790100, Palermo, Italy

**Keywords:** Transcatheter aortic valve implantation, Calcium score, Computed tomography angiography, Complications

## Abstract

**Purpose:**

The aim of our study was to evaluate the prevalence of early complications after Transcatheter Aortic Valve Implantation (TAVI) and their correlation with the Calcium Score (CS) of the aortic valve, aorta and ilio-femoral arteries derived from pre-procedural computed tomography (CT).

**Materials and methods:**

We retrospectively reviewed 226 patients (100 males, mean age 79.4 ± 6.7 years) undergoing 64-slice CT for pre-TAVI evaluation from January 2018 to April 2021. The population was divided into CS quartiles.

**Results:**

Overall, 173 patients underwent TAVI procedure, of whom 61% presented paravalvular leak after the procedure, 28% presented bleeding or vascular complications, 25% presented atrioventricular block, and 8% developed acute kidney injury. The prevalence of paravalvular leak and vascular complications was higher in the upper CS quartiles for aortic valve and ilio-femoral arteries.

**Conclusions:**

Aortic valve and vascular CS could help to predict post-TAVI early complications.

## Introduction

Transcatheter Aortic Valve Implantation (TAVI) is nowadays a valuable alternative treatment for severe aortic stenosis in patients with high operative risk or contraindications for surgical replacement of the aortic valve. In these patients, the benefit of TAVI significantly outweighs the risk of the procedure and leads to lower mortality at one-year follow-up [1–6].

Computed tomography (CT) has a crucial role in pre-TAVI planning for a correct patient selection before treatment [7, 8]. For TAVI procedural success it is essential to study patient's anatomy to ensure adequate planning of the operating route, choose the vascular access and the most suitable prosthesis [7, 8].

CT permits to reliably measure the size of the aortic root allowing a correct choice of the size of the prosthesis. It is possible to evaluate the morphology of the access path, the degree of vascular and aortic valve calcifications and any comorbidities [9–15].

Vascular and valvular calcifications have been associated with the risk of peri- and post-procedural bleeding and complications [16–22]. However, no objective method to quantify vascular calcium is currently used [16–22].

This retrospective study has the purpose to evaluate the correlation between the prevalence of early post-TAVI complications, such as vascular complications, hemorrhages, paravalvular leak, acute kidney injury (AKI), and pacemaker (PM) implantation [23–32], and the vascular (aorta and ilio-femoral arteries) and aortic valve Calcium Score (CS) derived from pre-TAVI CT imaging, in order to evaluate whether a quantitative assessment of vascular calcifications can help in the risk stratification and prediction of post-TAVI early complications.

## Materials and methods

### Study population

We retrospectively evaluated 226 consecutive patients (100 males and 126 females, mean age 79.4 ± 6.7) with severe aortic stenosis candidate for TAVI, undergoing thoraco-abdominal ECG-synchronized Computed Tomography Angiography (CTA) for pre-procedural evaluation between January 2018 and April 2021**.** These patients had contraindications to surgical valve replacement due to high operative risk calculated with the Euroscore system [6].

### CT calcium score and CT angiography protocols

All examinations were performed with 64-slice CT scanner (Ingenuity 64, Philips, Best, The Netherlands). The inclusion criteria for performing the 64-slice ECG-synchronized CTA were: low heart rate < 70 beats per minute (bpm); breath-hold during acquisition. The exclusion criteria were: previous adverse reactions to iodinated contrast medium, impaired respiratory and renal function, unstable clinical conditions. In case of bpm > 70 or irregular heart rate, patients were given oral or intravenous β-blocker therapy before scanning. Patients were scanned in the supine position during apnea performing a non-contrast sequential acquisition with prospective ECG-gating for CS followed by CTA scanning with retrospective ECG-gating, both extended from the clavicles to the proximal femoral portion (from aortic arch to femoral arteries) for the evaluation of the arterial access routes. The scanning and reconstruction parameters are summarized in Table 1.

**Table 1:** Scan and reconstruction parameters

*Scan parameters*	
Detectors (n)	64
Collimation (mm)	0.625
Beam energy (kV)	100
mAs	585
Rotation time (ms)	400
Scan time (s)	16
DLP (mGy*cm)	3838.25
*Reconstruction parameters*	
Slice thickness (mm)	1
Reconstruction increments (mm)	0.5
Field of View (mm)	650–750
Convolution filter/kernel	Medium-smooth and sharp
*Contrast media parameters*	
Volume (ml)	100
Flow rate (ml/s)	4
Iodine concentration (mgI/ml)	350–370
Bolus chaser	40 ml @ 4 ml/s
Venous access	Antecubital right

*DLP*—Dose length product

CT CS was performed with a standard protocol (3 mm thickness, 1.5 reconstruction interval). CTA was performed with 100 ml of non-ionic contrast agent (Iopromide 370 mg I/ml) administered intravenously at a rate of 4 ml/s, followed by 40 ml of saline at the same rate. The contrast agent was administered with an automatic dual-syringe injector connected with a 18G cannula into an antecubital vein of the right arm. The bolus tracking technique was used for scan synchronization. Acquired images were reconstructed during the mid-to-end diastolic phase, with a reconstruction window at 75% of the R-R interval and systolic phase at 25–35% of the RR interval for aortic annulus. When irregular heart rates, such as branch blocks or extrasystoles, were found, the temporal variability in the reconstruction phase was compensated manually with ECG editing. All images were acquired with a large field of view (which included the chest and abdomen up to the bifurcation of the femoral arteries) always reconstructed in each patient with the same parameters: 1 mm slice thickness, 0.5 mm increment, medium-smooth and sharp convolution kernels.

**Fig. 1:**
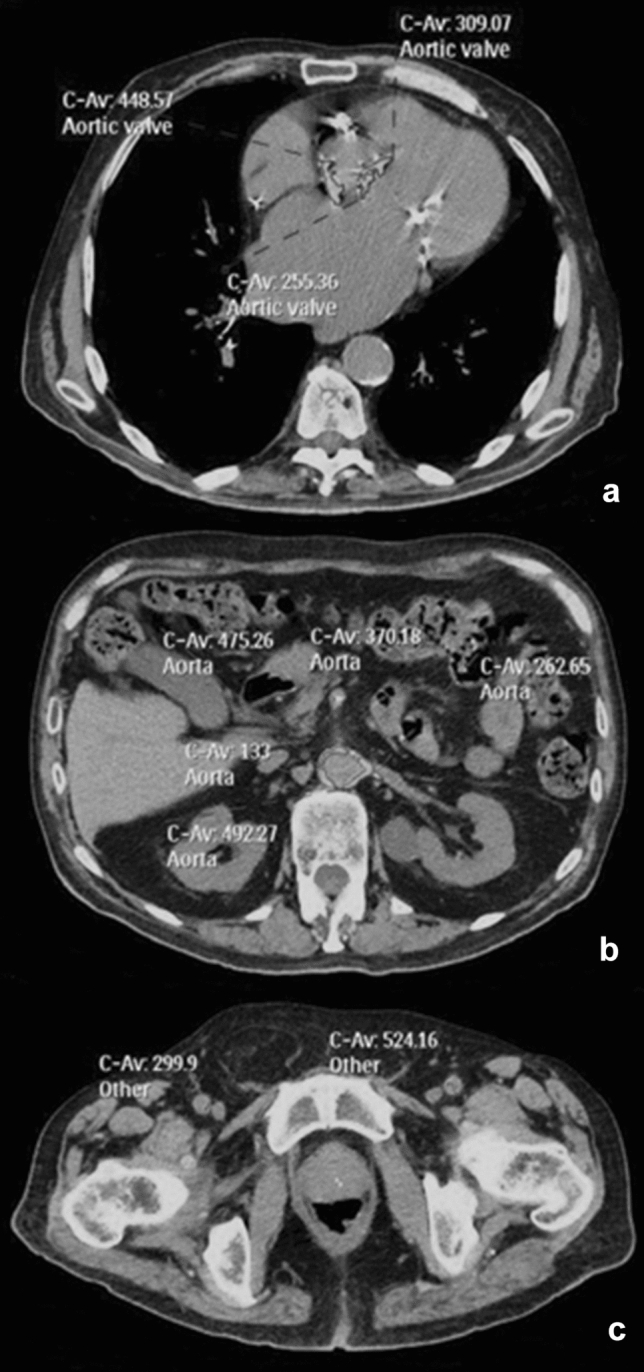
Vascular calcium score assessment: **a** aortic valve, 3329 AU; **b** aortic, 56,187 AU; **c** ilio-femoral, 12,320 AU

CS was calculated with a dedicated software (Philips HeartBeat) with Agatston score (in terms of Agatston Units, AU). The following anatomical districts were analyzed: aortic valve, aorta (from ascending aorta to aorto-iliac carrefour), and ilio-femoral arteries (Fig. 1).

The average DLP of the protocol was 3838.25 mGy*cm.

### Clinical follow-up

Follow-up was carried out with inpatient visit and medical records in hospitalized patients.

All patients were followed up for 1 month to record the following early post-procedural complications: paravalvular leaks, post-procedural AV-block, vascular complications, acute kidney injury (AKI) and stroke.

All patients were included in a clinical and instrumental six-monthly follow-up program for major adverse cardiac events (MACE).

### Statistical analysis

The results are expressed in terms of absolute values and percentages. The analysis was performed using the statistical options included in Microsoft Excel 365.

The population was divided into quartiles (Q) for aortic valve CS, aortic CS and ilio-femoral CS. The statistical difference between the 4 groups was analyzed for categorical variables, using χ^2^ square test and a value of p < 0.01 was considered significant. Student's T test was used to compare the CS averages of TAVI patients and patients excluded from the procedure. A value of p < 0.01 was considered significant.

Receiver operative characteristic (ROC) curves were used to identify a possible cut-off of aortic valve CS, aortic CS, ilio-femoral CS and overall CS.

## Results

### Calcium score characteristics

We examined 226 patients candidate for TAVI: 100 males and 126 females aged 51 to 94 years and mean age 79.4 ± 6.7 years. The mean aortic CS was 15,350.8 ± 12,078.5 (with a minimum score of 205 and a maximum of 73,055), the mean ilio-femoral CS was 6504.5 ± 6561.3 (with a minimum value of 0 and a maximum value of 37,164), the mean CS of the aortic valve was 3351.6 ± 2349.1 (with a minimum value of 0 and a maximum value of 13,146). The mean overall CS (aortic valve, aortic and ilio-femoral) was 25,042.5 ± 17,333.2 (with a minimum value of 570 and a maximum value of 100,599). The mean coronary CS was 800 ± 1060 (with a minimum score of 0 and a maximum of 5234). The patients characteristics are summarized in Table 2.

**Table 2:** Patients characteristics

Total patients (n)	226
Males n (%)	100 (44%)
Females n (%)	126 (56%)
Mean age (years)	79.4 ± 6.7
Aortic valve CS (mean ± SD)	3352 ± 2349
Aortic CS (mean ± SD)	15,351 ± 12,078
Ilio-femoral CS (mean ± SD)	6505 ± 6561
Aortic-femoral CS (mean ± SD)	21,826 ± 17,189
Overall CS (mean ± SD)	25,043 ± 17,333
Coronary CS (mean ± SD)	800 ± 1060
Hypertension n (%)	180 (80%)
Smoking n (%)	45 (19%)
Diabetes mellitus n (%)	78 (35%)
Dyslipidemia n (%)	95 (42%)
Obesity/overweight n (%)	28 (12%)
Familiarity CVD n (%)	25 (11%)
Underwent TAVI (n)	173

*CS*—Calcium Score, *TAVI*—Transcatheter aortic valve implantation, *CVD*—Cardiovascular disease

### TAVI procedures characteristics

Overall, 173 patients underwent TAVI (77 males and 96 females with mean age of 79 years ± 6.5) with retrograde transfemoral access in 168 patients (97%) and left trans-subclavian access in 5 patients. The valve prosthesis used were in order of frequency: Core valve 29 in 73 patients, Core valve 26 in 59 patients, Core valve 23 in 18 patients, Core valve 34 in 17 patients, Core valve 20 in 2 patients, Core valve 25 in 1 patient, and Abbott Portico valve in 3 patients, with diameters of 29, 27 and 23 mm, respectively. The remaining 53 patients were excluded from TAVI procedure after imaging and operative risk assessment, of which 36 had a high operative risk for inadequate operative route due to a high degree of calcification or tortuosity of the vessels; 17 were excluded for other comorbidities detected on CTA. In particular, 2 patients were excluded from TAVI for high risk of coronary occlusion due to coronary ostia-annulus distance inferior than 10 mm.

The 36 patients excluded for vascular calcifications, had a mean ilio-femoral CS of 11,807.4 ± 9411.5 (with a minimum value of 944 and maximum score of 37,164); the mean aortic CS was 23,351 ± 16,053.5 (with a minimum score of 983 and a maximum score of 67,697); the mean aortic valve CS was 3286.7 ± 2306.3 (with a minimum score of 183 and a maximum score of 9653). These patients had an average CS higher than patients undergoing TAVI, in particular the average ilio-femoral score was almost double the average value of the population subjected to TAVI. The comparison between the CS averages of the two groups was statistically significant for all the vascular districts considered. The results of the analysis are summarized in Table 3.

**Table 3:** Comparison between the CS averages of patients undergoing TAVI and patients excluded from TAVI

Anatomical district	Average CS undergoing TAVI	Average CS excluded from TAVI	p
Aortic valve	3575 ± 2365	3286 ± 2306	0.007
Aorta	13,760 ± 10,451	23,351 ± 16,053	0.004
Ilio-femoral axis	5320 ± 5116	11,807 ± 9411	0.0002

*TAVI*—Transcatheter aortic valve implantation, *CS*—Calcium score

In 173 patients undergoing TAVI, 105 (61%) underwent pre-surgery conventional coronary angiography for suspected coronary artery disease, among them: 69 (66%) did not present significant coronary stenosis, 15 (14%) had mild stenosis (> 50%), 14 (14%) moderate stenosis (< 70%), and 7 (6%) underwent percutaneous transluminal coronary angioplasty because of severe stenosis (> 70%). The prevalence of coronary stenosis of any degree and coronary stenosis > 50% was higher (p < 0.01) in subjects belonging to the third (Q3) and fourth quartiles (Q4) of coronary CS. Sixty-eight patients, with low risk for CAD and low CS score, did not underwent coronary angiography, because pre-TAVI CTA did not show any significant coronary stenosis. No MACE records were observed at six months follow-up in this subgroup.

### Post-TAVI complications

**Fig. 2:**
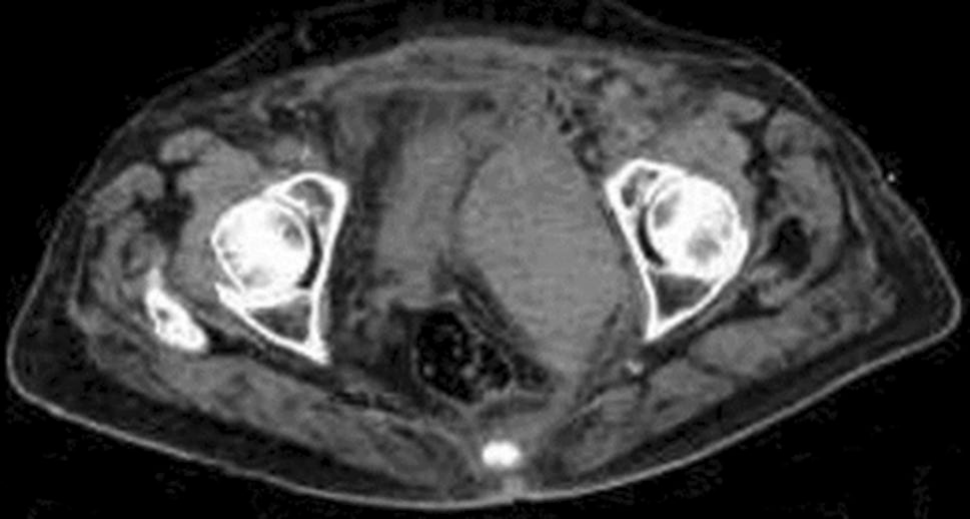
Large pelvic bleeding in a patient with an ilio-femoral CS of 20,837 AU

We observed the following prevalence of post-TAVI complications: 106 (61%) patients had paravalvular leaks, in particular 89 patients (52%) had minimal/mild valve leaks, 17 (10%) had moderate grade leaks; 43 patients (25%) were subjected to PM implantation for post-procedural AV-block; 49 patients (28%) had vascular complications, in particular 13 had bleeding from the access site including 4 with abdominal bleeding (Fig. 2), 5 patients presented thrombotic occlusion of the access site treated with thromboendarterectomy, 1 patient presented dissection of the femoral artery access site, and 43 patients underwent surgical revision of the access site due to complications or vascular closure device failure; 4 patients presented pericardial effusion; 14 patients developed AKI; one patient presented transient aphasia after the procedure. No coronary obstruction events were recorded.

**Fig. 3:**
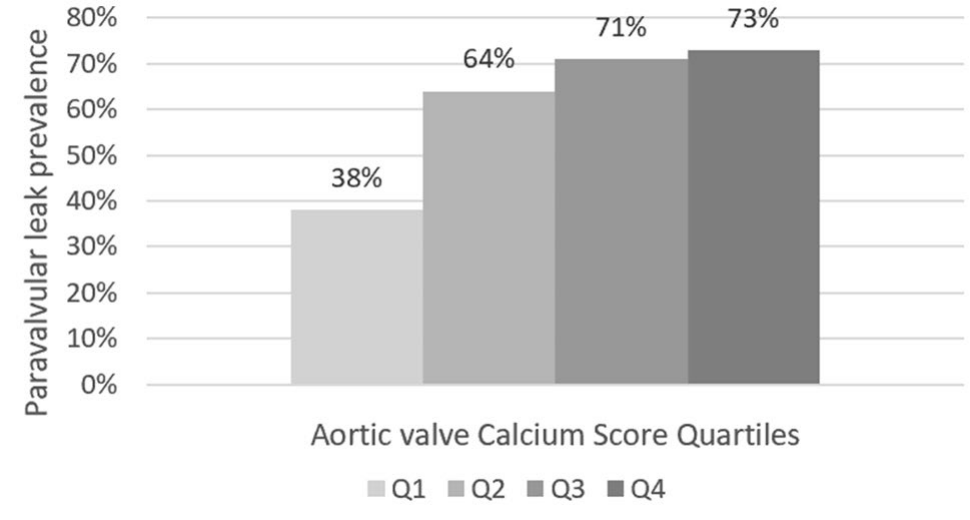
Paravalvular leak prevalence according to aortic valve CS quartiles: Q1 CS < 1909AU, Q2 CS < 3135AU, Q3 CS < 4574 AU, Q4 CS > 4574 AU

**Fig. 4:**
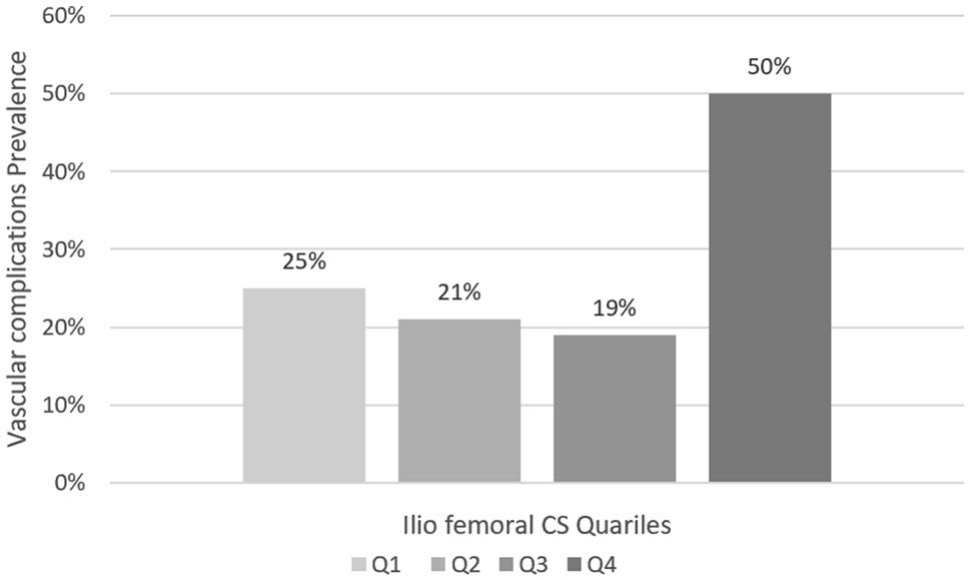
Vascular complications prevalence according to ilio-femoral CS quartiles: Q1 CS < 1906 AU, Q2 CS < 3692 AU, Q3 CS < 7988 AU, Q4 CS > 7988 AU

The Q3 and Q4 accounted higher prevalence for some complications. For the aortic valve CS, the prevalence of paravalvular leak was 73% in the Q3 and 71% in the Q4, while in the Q1 was 38%. In Q1 all patients had minimal or mild leaks. Among 106 patients with paravalvular leak, 71% of patients had a valvular CS value above 3135 AU (OR = 2.34, 95% CI 1.23–4.45). In addition, all subjects with moderate leaks had higher average scores, between the Q3 and Q4 (Fig. 3). In 49 subjects who presented vascular complications, 21 patients belonged to Q4 of ilio-femoral CS. The 50% of patients with ilio-femoral CS values above 7988 AU had vascular complications with an OR of 3.14 (95% CI 1.50–6.58), compared with 22% of patients with ilio-femoral CS values below 7988 AU (Fig. 4). In 13 subjects who suffered post-procedural femoral artery hemorrhage, 5 patients (38%) belonged to the Q4, representing the 17% of the population with a value of ilio-femoral CS above 7988 AU (OR = 2.93, CI 95% 0.89–9.68).

All the 4 patients who presented abdominal bleeding belonged to the Q3 and Q4.

No significant differences were found between the CS quartiles for the prevalence of other complications, such as PM implantation and AKI. No significant differences were found between quartiles of overall CS with respect to any complications. The difference in complication prevalence among the CS quartiles at the χ^2^ test was statistically significant (p = 0.006 for vascular complications of ilio-femoral CS and p = 0.009 for paravalvular leak of aortic valve CS). The results are summarized in Table 4.

**Table 4:** Prevalence of post-TAVI complications for CS quartiles

Post-TAVI complications	Q1 (%)	Q2 (%)	Q3 (%)	Q4 (%)	p
Para-valvular leak and aortic valve CS	38	64	71	73	0.009
Vascular complications and ilio-femoral CS	25	21	19	50	0.006
PM and aortic valve CS	27	21	28	23	0.86
AKI and aortic CS	4	11	10	10	0.68
Vascular complications and aortic CS	5	14	5	14	0.22
Any complication and overall CS	68	81	86	86	0.11

*TAVI*—Transcatheter aortic valve implantation, *CS*—Calcium score, *PM*—Pacemaker, *AKI*—Acute kidney injury

## ROC analysis

**Fig. 5:**
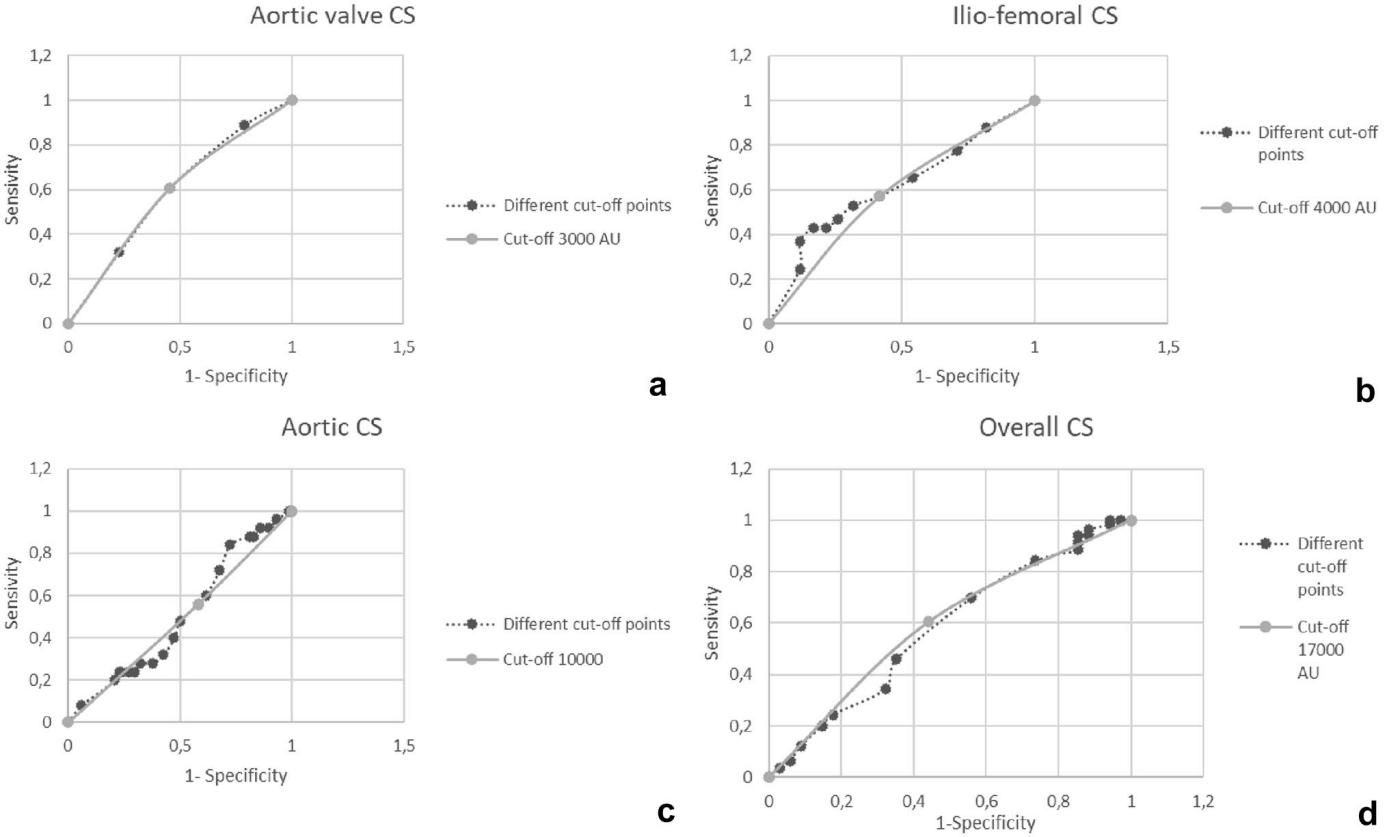
Receiver operative characteristic curves: **a** aortic valve CS cut-off of 3000 AU; **b** ilio-femoral CS cut-off of 4000 AU; **c** aortic CS cut-off of 10,000 AU; **d** overall CS (aortic valve, aortic and ilio-femoral CS) cut-off of 17,000

Results from the ROC analysis identified a cut-off of 3000 AU aortic valvular CS for paravalvular leak with an AUC of 0.82, showing 61% sensitivity and 55% specificity, a cut-off of 4000 AU ilio-femoral CS for vascular complications with an AUC of 0.82, showing 57% sensitivity and 58% specificity, a cut-off of 10,000 AU aortic CS for vascular complications and AKI with an AUC of 0.74, showing 56% sensitivity and 42% specificity, and a cut-off of 17,000 AU overall CS (aortic valvular, aortic and ilio-femoral) for any complication with an AUC of 0.83, showing 61% sensitivity and 56% specificity (Fig. 5). Lower cut-off points (10,000 AU, sensitivity 84%, specificity 27%, AUC 0.88) showed higher sensibility and AUC values, but lower specificity values.

## Discussion

In our study the higher prevalence of para-valvular leak and vascular complications in patients with higher CS values shows that valvular and vascular calcifications can be related to the onset of early post-TAVI complications. Therefore, CS could be used in the pre-TAVI imaging evaluation as a predictive factor to estimate the post-procedural complication risk. In particular, higher aortic valve CS is associated with a greater occurrence of paravalvular leaks. In patients with valvular CS values above 3135 AU the paravalvular leak occurred in more than 71% of patients versus 38% of patients with CS value below 1909 AU.

It is known that a subgroup of patients has limited benefit from TAVI and may be affected by several early complications [32]. In this setting, machine learning algorithms can optimize the prediction of the outcome [33]. Moreover, CT 3D-reconstruction model based on deep convolutional neural networks (DCNN) may provide better image quality and accuracy of traditional model-based iterative reconstructions [34].

In a study conducted by Leber et al. out of 68 TAVI patients with transfemoral access, the aortic valve CS was the only significant predictor for cardiac complications and was associated with the incidence and severity of post-procedural aortic regurgitation [17].

Another study conducted by Heansing et al. on 120 patients reported a significant association between the value of aortic valve CS and the paravalvular leak and tendency toward a higher incidence of new PM implants [18].

In our study, a higher ilio-femoral CS is also associated with a greater onset of vascular complications from the access site. For CS above 7988 AU, vascular complications occurred in 50% of patients, while for lower score a prevalence of about 20% was observed. The same correlation was highlighted in a study conducted by Oliveira et al. on 129 patients, undergoing transfemoral TAVI, that associates the CS of the aorto-ilio-femoral arteries with the risk of post-TAVI hemorrhagic complications, classified according to Valve Academic Research Consortium criteria, in which the value of vascular CS is an independent predictor of hemorrhagic events [16].

In our study, no significant difference was observed in the prevalence of other post-TAVI complications such as the onset of AV-block, consequent PM implantation, and the onset of AKI. Furthermore, it was not possible to evaluate the correlation with the onset of cerebral ischemic events, as only one episode of transient post-procedural aphasia was detected. Moreover, no cases of myocardial ischemia determined by obstruction of the coronary ostia have been recorded.

Some limitations occur in our study. First, the cross-sectional design and the small sample of the study may limit the value of the results. However, the correlation observed between CS and the onset of paravalvular leaks and vascular complications consolidates the close association found in other studies [16–18]. Second, we employed a 64-slice CT scanner with a high radiation dose acquisition protocol. Advanced CT scanners with faster acquisition and protocols allow performing pre-TAVI CTA with lower contrast media volume, lower radiation dose and better image quality [35].

Our results highlight the need to quantitatively evaluate the degree of vascular and aortic valve calcifications by assessing the CS in the pre-TAVI CT imaging. Such assessment could be useful to select the size of the valve, reduce the risk of paravalvular leak and evaluate the degree of femoral vascular calcification, allowing to choose an alternative access route in the presence of high CS. Our results should be confirmed in a larger population on a multicenter basis. In particular, other studies are necessary to develop a risk stratification system based on vascular and valvular CS cut-off values, similarly to coronary CS employed in the risk stratification of coronary artery disease.

## Conclusions

The aortic valve, aortic and ilio-femoral arteries CS in pre-TAVI CT imaging could be an additional tool to reconsider the indication to TAVI in elderly patients. Vascular CS parameters could be integrated into the pre-TAVI assessment for risk stratification and patient selection. Furthermore, advanced CT scanners and deep learning reconstruction models could support the use of vascular CS in patients undergoing TAVI.

## References

[1] Lehmkuhl L, Foldyna B, Haensig M (2013). Role of preprocedural computed tomography in transcatheter aortic valve implantation. Rofo.

[2] Vahanian A, Beyersdorf F, Praz F (2022). 2021 ESC/EACTS Guidelines for the management of valvular heart disease. Eur Heart J.

[3] Nishimura RA, Otto CM, Bonow RO (2014). 2014 AHA/ACC Guideline for the Management of Patients With Valvular Heart Disease: a report of the American College of Cardiology/American Heart Association Task Force on Practice Guidelines. Circulation.

[4] Santoro G, Vitali E, Tamburino C (2010). Transcatheter aortic valve implantation for patients with aortic stenosis: a scientific statement from the Italian Federation of Cardiology (FIC) and the Italian Society for Cardiac Surgery (SICCH). G Ital Cardiol (Rome).

[5] Tarantini G, Esposito G, Musumeci G (2018). Updated SICI-GISE position paper on institutional and operator requirements for transcatheteraortic valve implantation. G Ital Cardiol (Rome).

[6] Roques F, Michel P, Goldstone AR, Nashef SA (2003). The logistic EuroSCORE. Eur Heart J.

[7] La Grutta L, Toia P, Grassedonio E (2020). TAVI imaging: over the echocardiography. Radiol Med.

[8] Achenbach S, Delgado V, Hausleiter J (2012). SCCT expert consensus document on computed tomography imaging before transcatheter aortic valve implantation (TAVI)/transcatheter aortic valve replacement (TAVR). J Cardiovasc Comput Tomogr.

[9] Wuest W, Anders K, Schuhbaeck A (2012). Dual source multidetector CT-angiography before Transcatheter Aortic Valve Implantation (TAVI) using a high-pitch spiral acquisition mode. Eur Radiol.

[10] Jurencak T, Turek J, Kietselaer BL (2015). MDCT evaluation of aortic root and aortic valve prior to TAVI. What is the optimal imaging time point in the cardiac cycle?. Eur Radiol.

[11] Tops LF, Wood DA, Delgado V (2008). Noninvasive evaluation of the aortic root with multislice computed tomography implications for transcatheter aortic valve replacement. JACC Cardiovasc Imaging.

[12] Leipsic J, Gurvitch R, Labounty TM (2011). Multidetector computed tomography in transcatheter aortic valve implantation. JACC Cardiovasc Imaging.

[13] Gurvitch R, Wood DA, Leipsic J (2010). Multislice computed tomography for prediction of optimal angiographic deployment projections during transcatheter aortic valve implantation. JACC Cardiovasc Interv.

[14] Hayashida K, Lefèvre T, Chevalier B (2011). Transfemoral aortic valve implantation new criteria to predict vascular complications. JACC Cardiovasc Interv.

[15] Harris BS, De Cecco CN, Schoepf UJ (2015). Dual-source CT imaging to plan transcatheter aortic valve replacement: accuracy for diagnosis of obstructive coronary artery disease. Radiology.

[16] Félix-Oliveira A, Campante Teles R, Ferreira AM (2018). Vascular calcium score: new imaging tool for prediction of major and life-threatening bleeding events in trans-femoral TAVI. Eur Heart J.

[17] Leber AW, Kasel M, Ischinger T (2013). Aortic valve calcium score as a predictor for outcome after TAVI using the CoreValverevalving system. Int J Cardiol.

[18] Haensig M, Lehmkuhl L, Rastan AJ (2012). Aortic valve calcium scoring is a predictor of significant paravalvular aortic insufficiency in transapical-aortic valve implantation. Eur J Cardiothorac Surg.

[19] Ribeiro HB, Webb JG, Makkar RR (2013). Predictive factors, management, and clinical outcomes of coronary obstruction following transcatheter aortic valve implantation: insights from a large multicenter registry. J Am Coll Cardiol.

[20] Stähli BE, Nguyen-Kim TD, Gebhard C (2015). Prosthesis-specific predictors of paravalvular regurgitation after transcatheter aortic valve replacement: impact of calcification and sizing on balloon-expandable versus self-expandable transcatheter heart valves. J Heart Valve Dis.

[21] Rodríguez-Palomares JF, Evangelista Masip A (2016). Aortic Calcium Score and Vascular Atherosclerosis in Asymptomatic Individuals: Beyond the Coronary Arteries. Rev Esp Cardiol.

[22] Sandfort V, Bluemke DA (2017). CT calcium scoring. History, current status and outlook. Diagn Interv Imaging.

[23] Wendler O, Thielmann M, Schroefel H (2013). Worldwide experience with the 29-mm Edwards SAPIEN XT™ transcatheter heart valve in patients with large aortic annulus. Eur J Cardiothorac Surg.

[24] Johansson M, Nozohoor S, Kimblad PO (2011). Transapical versus transfemoral aortic valve implantation: a comparison of survival and safety. Ann Thorac Surg.

[25] Ussia GP, Barbanti M, Sarkar K (2012). Transcatheter aortic bioprosthesis dislocation: technical aspects and midterm follow-up. EuroIntervention.

[26] Bleiziffer S, Ruge H, Hörer J (2010). Predictors for new-onset complete heart block after transcatheter aortic valve implantation. JACC Cardiovasc Interv.

[27] Ussia GP, Barbanti M, Petronio AS, Tarantini G (2012). Transcatheter aortic valve implantation: 3-year outcomes of self-expanding CoreValve prosthesis. Eur Heart J.

[28] Eltchaninoff H, Prat A, Gilard M, Leguerrier A (2011). Transcatheter aortic valve implantation: early results of the FRANCE (FRench Aortic National CoreValve and Edwards) registry. Eur Heart J.

[29] Hynes BG, Rodés-Cabau J (2012). Transcatheter aortic valve implantation and cerebrovascular events: the current state of the art. Ann N Y Acad Sci.

[30] Saia F, Ciuca C, Taglieri N (2013). Acute kidney injury following transcatheter aortic valve implantation: incidence, predictors and clinical outcome. Int J Cardiol.

[31] Nuis RJ, Rodés-Cabau J, Sinning JM (2012). Blood transfusion and the risk of acute kidney injury after transcatheter aortic valve implantation. Circ Cardiovasc Interv.

[32] Van Mieghem NM, Tchetche D, Chieffo A (2012). Incidence, predictors, and implications of access site complications with transfemoral transcatheter aortic valve implantation. Am J Cardiol.

[33] Lopes RR, van Mourik MS, Schaft EV (2019). Value of machine learning in predicting TAVI outcomes. Neth Heart J.

[34] Zhang K, Gao Y, Lv J, Li J, Liu J (2022). Artificial intelligence-based spiral CT 3D reconstruction in transcatheter aortic valve implantation. Comput Math Methods Med.

[35] Dankerl P, Hammon M, Seuss H (2017). Computer-aided evaluation of low-dose and low-contrast agent third-generation dual-source CT angiography prior to transcatheter aortic valve implantation (TAVI). Int J Comput Assist Radiol Surg.

